# Core functions of primary care in Amathole District, South Africa: a descriptive study

**DOI:** 10.3399/BJGPO.2024.0141

**Published:** 2025-07-02

**Authors:** Robert Mash, Jenny Nash

**Affiliations:** 1 Division of Family Medicine and Primary Care, Stellenbosch University, Stellenbosch, South Africa; 2 Eastern Cape Department of Health, District Clinical Specialist Team, Bisho, South Africa

**Keywords:** facility access, health care quality assessments, primary care, South Africa, health services accessibility

## Abstract

**Background:**

Strengthening primary care is a priority globally and for the South African health system. The current measurement tools in South Africa do not assess the core functions of primary care: first contact access, comprehensiveness, coordination, continuity, and person-centredness. A regional version of the Primary Care Assessment Tool (PCAT) has recently been validated and can measure these core functions.

**Aim:**

To field test the regional PCAT and measure the core functions of primary care performance.

**Design & setting:**

A descriptive cross-sectional survey in Amathole District, South Africa.

**Method:**

Data were collected from 386 randomly selected patients from 40 clinics and six subdistricts. Data were collected using the REDCap mobile application and analysed in the Statistical Package for Social Sciences (version 27).

**Results:**

The median primary care score was 3.3 (interquartile range [IQR] 3.2–3.5) where a score ≥3 was seen as acceptable performance and ≥3.5 as good. Person-centredness, coordination, and utilisation were all scored as good (4.0, IQR 4.0–4.0). Comprehensiveness (3.3, IQR 2.9–3.6) and continuity (3.2, IQR 3.1–3.6) were scored as acceptable. Access to care was scored as poor (1.7, IQR 1.0–2.9). There were significant differences in primary care scores between subdistricts. Patients with a worse health status or chronic condition gave lower scores. The most affluent and the poorest groups also gave lower scores.

**Conclusion:**

The district needs to focus on improving access to care as well as some aspects of comprehensiveness, continuity, and coordination. The newly validated regional PCAT can provide the district with novel information for performance management and improvement.

## How this fits in

District health information systems in South Africa measure inputs, outputs, and outcomes, but not the core functions of quality primary care: first contact access, continuity, coordination, comprehensiveness, and person-centredness. A new sub-Saharan version of the Primary Care Assessment Tool (PCAT) was used for the first time in this study to measure the core functions of primary care. The core functions of primary care performance have not, to the authors’ knowledge, been previously measured in the Eastern Cape of South Africa. This study showed that first contact access was poor, comprehensiveness and continuity were acceptable, while coordination, utilisation, and person-centredness were performing well.

## Introduction

Strengthening primary health care (PHC) is a global priority.^1^ Ever since 1994 and the end of apartheid in South Africa, implementing high quality PHC has been a policy goal.^2^ Health reforms have focused on the introduction of community health worker teams, family physicians, district specialist clinical teams, and enhanced school health services. In 2024, the South African Government signed a Bill to implement national health insurance, which will require the provision of high quality primary care.

The World Health Organization (WHO) has introduced a new measurement framework for PHC.^3^ The South African health system has several tools to measure the performance of primary care. The Ideal clinic programme introduced a range of norms and standards.^4^ All primary care facilities are evaluated annually for their progress towards achieving ’ideal’ status. These norms and standards focus primarily on measuring health system inputs and service delivery processes. In the Eastern Cape, where this study is located, the proportion of clinics with ’ideal’ status increased from 2% in 2016 to 63% in 2024.^4^

The other major source of information is the District Health Information System, and the key indicators are presented each year in the District Health Barometer.^5^ These mostly measure key outputs of service delivery related to diseases or programmes and some outcomes. In the Amathole District, the focus of this study, the district is in the highest quartile (out of a total of 52 districts) for effective antiretroviral treatment coverage and cervical cancer screening, but in the lowest quartile for antenatal first visit coverage and teenage pregnancies.

When reviewing the WHO measurement framework, the aspect that is not measured is the core functions of primary care. These are regarded as essential outputs for the quality of primary care: first contact access, comprehensiveness, continuity, coordination, and person-centeredness.^3^ Researchers in sub-Saharan Africa have recently validated a regional version of the Primary Care Assessment Tool (PCAT) that can measure these core functions.^6^ Although country versions of the PCAT have previously been used in South Africa, Malawi, Kenya, and Uganda, the regional version has not yet been field tested.^7–10^ To the authors’ knowledge, this study is the first to test the regional PCAT in the Amathole District of the Eastern Cape in South Africa. In addition, the PCAT has never been used to evaluate primary care performance in this province. The aim of this study, therefore, was to evaluate the core functions of primary care using the regional PCAT in the Amathole District.

## Method

### Study design

A descriptive cross-sectional survey of primary care facilities.

### Setting

The Amathole District is situated in the Eastern Cape, one of the poorest rural provinces in South Africa. The Xhosa population is estimated at 778 884, with a higher proportion of children, adolescents, and older adults when compared with the national population.^5^ Working age adults often seek work in other provinces. There are six sub-districts (technically three are categorised as local municipalities) that offer services through a network of small primary care clinics (Table 1). Clinics are usually staffed by nurse practitioners and may have a doctor visiting. Some clinics are remote and can only be accessed by four-wheel drive vehicles. Subdistricts may also have mobile clinics or larger community health centres that include a doctor(s), dentist, pharmacist, and radiographer.

**Table 1.** Primary care facilities and subdistricts in Amathole District

Subdistrict	Community health centre, *n*	Clinic, *n*	Mobile, *n*	Total, *n*	Primary care headcount, *n*
Raymond Mhlaba	1	36	4	41	396 759
Ngqushwa	0	22	3	25	184 645
Mnquma	1	28	3	32	539 295
Mbhashe	3	29	6	38	593 940
Great Kei	0	4	0	4	83 634
Amahlathi	0	25	7	32	308 385
Total	5	144	23	172	2 106 658

### Study population, sample size and selection

For a population of 2 106 658 (annual primary care headcount, see Table 1) with 95% confidence intervals (CIs), 5% margin of error, and 50% scoring any given domain ≥3, a sample of 385 would be considered adequate.^11^ An intended sample of 400 was stratified between subdistricts according to the primary care headcount. The allocated sample was then divided by 10 to give the number of facilities to be sampled per subdistrict — assuming an average of 10 patients per facility. It was only feasible to collect data from 40 facilities given the resources, distances, and travel conditions. The required number of facilities were then randomly selected from the list (excluding mobile clinics) for each subdistrict and the final sample required from each facility stratified by headcount.

### Data collection

The sub-Saharan version of the Primary Care Assessment Tool (PCAT-SSA) was used. The PCAT-SSA was validated for the region to measure the core functions of primary care. Each function was measured by several items on a Likert scale from definitely not (1) to definitely (4) and are defined in Table 2. In addition, the tool measured affiliation to the facility, self-reported health status, and sociodemographic variables.

**Table 2.** Definitions of the core functions as measured by PCAT-SSA

Core function	Definition
**First contact access**	How easy is it for users to access primary care as the first place that they go when looking for assistance with a health issue.
**Continuity**	Informational continuity focuses on whether an up-to-date medical record is available when they attend primary care; relational continuity focuses on whether they develop a relationship of trust with and are known by their primary care provider.
**Coordination**	Parallel coordination focuses on coordination between teams within the PHC level of care; sequential coordination focuses on coordination between teams in different levels of care.
**Comprehensiveness**	Focuses on whether primary care covers the lifespan, the burden of disease, and includes health promotion, disease prevention, treatment, palliation, and rehabilitation.
**Person-centredness**	Focuses on whether care incorporates both the biomedical and users’ perspectives on the illness, and whether care focuses on the person and not just the disease.

PCAT-SSA = sub-Saharan version of the Primary Care Assessment Tool. PHC = primary health care.

The questionnaire was forward translated by a first language isiXhosa speaker at Stellenbosch University and then back translated by the research assistants. Three newly qualified isiXhosa speaking female doctors were research assistants. They had no prior relationship with the facilities and did not present themselves as doctors. They understood the concepts and administered the questionnaire to patients in isiXhosa.

The research assistants used systematic random sampling to select the patients. Patients were interviewed as they exited the facility and data captured on the REDCap mobile application. Any adult patient (aged ≥18 years) could be selected if they had attended the facility at least three times.

### Data analysis

Data were exported from REDCap into the Statistical Package for Social Sciences (version 27) and analysed by RM. An average score for each of the core functions was calculated from the Likert scale (1–4) for all associated items. A primary care score was calculated from the average of the core function scores. Numerical scores were described as means and standard deviations (SDs) or medians and interquartile ranges (IQRs), depending on their distribution. Numerical scores were also categorised into the proportion that scored <3 versus ≥3. A score of ≥3 was seen as indicating at least an acceptable quality of care.

Affiliation was calculated from responses to three items that assessed whether the patient attended another facility and, if so, which facility knew them best. The self-reported health status and sociodemographics were mostly categorical data and reported as frequencies and percentages.

Inferential analysis looked for any associations between subdistricts, health status, and sociodemographic variables with the primary care score. Nominal categorical data were analysed using analysis of variance and *post-hoc* Bonferroni tests were used to determine significant relationships (*P*<0.05). Binary categorical data were analysed by the independent samples *t*-test, while age was analysed using Spearman’s correlation.

## Results

Complete data were obtained for 386 patients and their characteristics are shown in Table 3. The mean age was 49.1 years (SD 17.5), 298 (77.2%) were women, and 384 (99.5%) spoke isiXhosa. Responders made a median of 12.0 (IQR 8.0–14.7) visits to the facility over the previous 2 years, most had been a patient for ≥5 years (68.6%), and 77.1% had a strong affiliation. Responders said that they attended the facility because it was geographically close (60.1%) and offered a good quality of care (64.0%). The majority described their health as good to excellent (73.0%) and 23.0% had a chronic condition.

**Table 3.** Characteristics of responders

Characteristic	*n* (%)
**Affiliation to the primary care facility (*N* = 385**)	
Strong	297 (77.1)
Moderate	81 (21.0)
Weak	7 (1.8)
**How long have you been a patient at the facility? (*N* = 385)**	
<1 year	13 (3.4)
1–2 years	43 (11.2)
3–4 years	63 (16.4)
≥5 years	264 (68.6)
Unsure	2 (0.5)
**Why did you choose this facility? (*N* = 386)**	
Because it is close to where I live or work	232 (60.1)
Because I can afford the fees	3 (0.8)
Because it offers good quality health care	247 (64.0)
Because I am registered here and not allowed to go elsewhere	10 (2.6)
Because my insurance covers me here	0 (0.0)
Because I was referred here by another facility	6 (1.6)
Other	8 (2.1)
**What is your home language? (*N* = 386)**	
Xhosa	384 (99.5)
Afrikaans	1 (0.3)
English	1 (0.3)
**Which of the following best describes your work situation? (*N* = 386)**	
Employed full time	45 (11.7)
Employed part time	14 (3.6)
Self-employed (informal)	23 (6.0)
Self-employed (formal)	4 (1.0)
Student	24 (6.2)
Home maker	7 (1.8)
Retired	115 (29.8)
Unemployed	154 (39.9)
**Which of the following best describes your schooling? (*N* = 386)**	
Did not complete primary school	68 (17.6)
Completed primary school	39 (10.1)
Did not complete high school	166 (43.0)
Completed high school	85 (22.0)
Completed tertiary education	26 (6.7)
Refuse to answer	2 (0.5)
**Do you have piped water? (*N* = 386)**	
Yes, inside my house	44 (11.4)
Yes, inside my yard	175 (45.3)
Yes, nearby	63 (16.3)
No	104 (26.9)
**Do you have electricity in your home? (*N* = 384)**	
Yes	364 (94.8)
**Which of the following best describes your home? (*N* = 384)**	
Structure made of brick or concrete blocks	284 (74.0)
Structure made of wood, zinc, or mud	100 (26.0)
Refuse	1 (0.3)
**Is anyone employed in your household? (*N* = 385)**	
Yes	193 (50.1)
**Do you have water-borne sewage (i.e. a toilet)? (*N* = 385)**	
Yes, inside my house	51 (13.2)
Yes, inside my yard	190 (49.4)
Yes, nearby	2 (0.5)
No	141 (36.6)
**Would you say your health status is: (*N* = 385)**	
Excellent	51 (13.2)
Very good	135 (35.1)
Good	95 (24.7)
Fair	94 (24.4)
Poor	10 (2.6)
**Do you have a chronic condition? (*N* = 382)**	
Yes	88 (23.0)

Patients had little education as 70.7% did not finish high school. A large number were unemployed (39.9%) or retired (29.8%), and only 11.7% said they had full-time employment. Only one-half of all households had anyone employed. A substantial proportion were living in informal dwellings (26.0%), only 11.4% had potable water, and only 13.2% a toilet inside their house. The majority (94.8%) said they had electricity (Table 3).

Table 4 presents the scores for the core functions of primary care and overall primary care score. The scores were interpreted as very poor performance (<1.5), poor (1.5 to 2.4), acceptable (2.5 to 3.4), and good (≥3.5). Access to primary care was poor, comprehensiveness and continuity were acceptable, while person-centredness, coordination, and utilisation were good. The overall primary care score was acceptable.

**Table 4.** Core functions of primary care

Function	Median score (IQR)	Scoring ≥**3,** *n* (%)	Interpretation
Primary care (*N* = 333)	3.3 (3.2–3.5)	308 (92.5)	Acceptable
Access (*N* = 385)	1.7 (1.0–2.9)	96 (24.9)	Poor
Utilisation (*N* = 384)	4.0 (4.0–4.0)	384 (100.0)	Good
Comprehensiveness (*N* = 344)	3.3 (2.9–3.6)	259 (75.3)	Acceptable
Continuity (*N* = 379)	3.2 (3.1–3.6)	332 (87.6)	Acceptable
Person-centeredness (*N* = 380)	4.0 (4.0–4.0)	368 (96.8)	Good
Coordination (*N* = 386)	4.0 (4.0–4.0)	366 (94.8)	Good

IQR = interquartile range.

Looking at the results for individual items, the key issues leading to a poor score for access were the opening times of primary care facilities (no services in the evening or weekends) and the inability to obtain help out-of-hours (no one to call and no one to visit you at home). For comprehensiveness, the areas of concern were dental care, counselling for social problems, treatment of minor injuries and trauma, assessment of hearing and eyesight, minor surgery, help with disabilities, palliative care, and assisting the older adult. For continuity, the main issue was not seeing the same primary care provider. For coordination, the main issue was that the primary care provider did not make the appointment for the first visit to the hospital (data not shown).

Table 5 presents the relationship of subdistricts, health status, and sociodemographic factors to the overall primary care score. Subdistricts significantly differed in performance with better scores in the more rural and remote areas. Patients who rated their health as very good rated primary care higher than those who rated their health good or fair, while those with a chronic condition rated primary care as significantly lower.

**Table 5.** Associations between subdistricts, health status, and sociodemographic factors and mean primary care score

Variable	Mean primary care score (95% CI)	*P* value
**Subdistrict**		
Mnquma	3.3 (3.2 to 3.3)	<0.001
Mbhashe	3.5 (3.4 to 3.5)	
Amahlathi	3.3 (3.2 to 3.3)	
Ngqushwa	3.4 (3.3 to 3.5)	
Great Kei	3.1 (3.0 to 3.2)	
Raymond Mhlaba	3.4 (3.3 to 3.4)	
**Gender**		
Man	3.3	0.103
Woman	3.4	
Mean difference	-0.1 (-0.1 to 0.0)	
**Education**		
Did not complete primary school	3.4 (3.3 to 3.5)	0.323
Completed primary school	3.4 (3.3 to 3.5)	
Did not complete secondary school	3.3 (3.3 to 3.4)	
Completed secondary school	3.4 (3.3 to 3.4)	
Completed tertiary education	3.3 (3.2 to 3.4)	
**Type of house**		
Structure made of brick or concrete blocks	3.4	0.018
Structure made of wood, zinc, or mud	3.3	
Mean difference	0.1 (0.0 to 0.1)	
**Employment in household**		
Yes	3.3	0.459
No	3.4	
Mean difference	-0.0 (-0.1 to 0.0)	
**Employment of patient**		
Employed full time	3.3 (3.3 to 3.3)	0.014
Employed part time	3.3 (3.1 to 3.5)	
Self-employed (informal)	3.4 (3.2 to 3.5)	
Self-employed (formal)	3.5 (3.2 to 3.7)	
Student	3.2 (3.0 to 3.3)	
Home maker	3.4 (3.2 to 3.6)	
Retired	3.3 (3.3 to 3.4)	
Unemployed	3.4 (3.3 to 3.5)	
**Piped water**		
Yes, inside house	3.2 (3.1 to 3.3)	<0.001
Yes, in yard	3.4 (3.4 to 3.5)	
Yes, nearby	3.4 (3.3 to 3.5)	
No	3.2 (3.2 to 3.3)	
**Electricity**		
Yes	3.3	0.308
No	3.3	
Mean difference	0.1 (-0.1 to 0.2)	
**Toilet**		
Yes, inside house	3.2 (3.1 to 3.3)	
Yes, in yard	3.5 (3.4 to 3.5)	
Yes, nearby	3.9 (3.3 to 4.5)	
No	3.2 (3.2 to 3.3)	
**Health status**		
Excellent	3.4 (3.3 to 3.4)	<0.001
Very good	3.5 (3.4 to 3.5)	
Good	3.3 (3.2 to 3.3)	
Fair	3.3 (3.2 to 3.3)	
Poor	3.3 (3.1 to 3.4)	
**Chronic condition**		
Yes	3.2	<0.001
No	3.4	
Mean difference	-0.2 (-0.3 to -0.2)	

Age (*r* = 0.04; *P* = 0.453; data not shown), gender, level of education, and employment in the household were not related to the primary care score (Table 5). People living in more informal dwellings and without running water or a toilet in the dwelling gave a lower primary care score. However, people with running water and toilets inside their dwelling also gave a lower score when compared with those who had access nearby. Students gave significantly lower primary care scores than those who were unemployed.

## Discussion

### Summary

Patients rated primary care performance as acceptable. Person-centredness, coordination, and utilisation were rated as good. Continuity and comprehensiveness were seen as acceptable. Relational continuity was weak, while informational continuity was stronger. Service delivery for dental care, social problems, minor injuries, minor surgery, people with disabilities, hearing and eye care, palliative care, and care for older people undermined the score for comprehensiveness. Access was scored poorly as people could not access any primary care on evenings or weekends, neither at their facility nor in their home or via mobile phone. Nevertheless, affiliation to the facilities was high.

Patients who had worse health or a chronic condition scored primary care performance lower. There was also unequal performance between subdistricts, with more rural subdistricts performing better. There was some evidence that both the poorest and the most affluent sections of the community rated performance lower.

### Strengths and limitations

To the authors’ knowledge, this is the first study to use the PCAT-SSA, with a focus on the core functions of primary care. The study obtained the required sample size and followed the intended sampling template. The results, therefore, should be generalisable to the district. The results were more positive than expected but there did not appear to be any social desirability bias with the research assistants who were not identified as doctors. They were perceived as young Xhosa speaking women and external to the clinics. The findings came from three different assistants and 40 different facilities across the whole district, which should have minimised any such bias. It might have been helpful to triangulate the patients’ scores with those of the managers or healthcare workers to get a more complete picture of the core functions. The predominance of women attending primary care is consistent with patterns elsewhere,^12^ and may also reflect migrant labour issues, with many working-age men in the Eastern Cape looking for work in other provinces.

### Comparison with existing literature

Table 6 compares Amathole with other studies that used PCAT in South Africa, Kenya, and Uganda.^9,12,13^ The study from South Africa combined data from four other provinces, but not the Eastern Cape. By comparison, Amathole scored slightly higher for all the core functions, apart from access. The study from Kenya was in metropolitan private sector primary care facilities, while the Ugandan study was from rural public sector primary care facilities. These previous studies used country level adaptations of the PCAT that differed slightly in the number of items and constructs. Again, Amathole performed better on all core functions, apart from access. It is difficult to attribute reasons for this, but two possible factors are active participation in the Ideal clinic programme and a well-functioning district clinical specialist team, led by a family physician, that consistently focused on supportive supervision and quality improvement.

**Table 6.** Comparison of performance with studies from South Africa,^12^ Kenya,^9^ and Uganda^13^

	Mean score		Median score	
Core function	South Africa	Kenya	Uganda	Amathole
Primary care	3.1	2.6	3.0	3.3
Access	2.5	2.3	2.6	1.7
Utilisation	3.4	3.1	3.0	4.0
Comprehensiveness	3.2	2.1	3.0	3.3
Continuity	2.9	2.8	2.6	3.2
Person-centredness	—	—	3.5	4.0
Coordination	3.3	2.9	2.3	4.0

Access to care can be related to geography, finances, and organisational issues.^14^ Many of the facilities in Amathole are in remote rural areas where roads are poor and people may need to walk to the facility. In South Africa there are no direct fees to use primary care, although costs may be incurred in travel or time off work. Many employers have a ‘no work, no pay’ approach, so working people prefer to access care outside of working hours. This could also be true of students who are committed during the day. Unfortunately, in Amathole there are no primary care services available outside of working hours and people with emergencies try to access care at the district hospital. The lack of access to primary care has been noted as a key factor in people with non-emergencies attending emergency departments.^15^ It should also be noted that only 3% of hospitals in the Eastern Cape were rated as ’ideal’ in 2023.^4^ Many of the communities had community health workers; however, it did not appear that people saw them as a resource outside of working hours. The public sector in South Africa has not developed any telehealth services that could support people afterhours.

Continuity is related to lower mortality and reduced hospitalisation and use of out-of-hours services.^16^ Such continuity is still an aspirational goal for South African primary care. Although coordination scored well, referral to specialist care required a doctor, and doctors were mostly available at the district hospitals. Therefore, primary care was unable to directly refer patients to specialists.

### Implications for practice

The most important issue to address is access to primary care outside of normal working hours. Although the health centres are open 24 hours a day they do not provide access to a doctor and there were only five health centres across the whole district. Extended hours on evenings and weekends might assist students and employed patients in some areas. Consideration should be given to help from community health workers out-of-hours and to the use of telehealth.

The comprehensiveness of primary care needs to be addressed and linkage to care for services related to dentistry, hearing loss, visual loss, and disability. Primary care nurses might need training in some issues such as social problems and linking people to social services. Palliative care may be an additional area for training as well as assisting older adults. Minor injuries and minor surgery might require better access to doctors at the primary care level. Even at the district hospital level some of these services may be problematic.

Improving the coordination of care for patients needing referral to specialist services should be streamlined. Ideally, clinics should be supported by visiting primary care doctors, or nurses should have access to specialist referral via digital solutions such as the Vula application.^17^ The Vula application allows a nurse to present the patient and receive feedback from the specialist via their mobile phone. An appointment can then be made without necessarily requiring an additional visit to the district hospital.

The model of care should be re-visited to enable more relational continuity with the primary care provider. This may be a particular issue in larger facilities with multiple providers.

The user version of the regional PCAT worked well in this setting and could be completed within 10 to 15 minutes. Creating health workers and manager versions might be useful to allow triangulation of results in the analysis.

Primary care performance in the Amathole District was acceptable and person-centredness, coordination, and utilisation were strengths. Comprehensiveness and continuity of care were acceptable but could be strengthened further. Access was poor and needs attention within the performance management process. The new regional version of the PCAT was easy to use and provided novel information on performance.

## Data

The dataset relied on in this article is available from the corresponding author on reasonable request.
